# *S*-(+)-Carvone, a Monoterpene with Potential Anti-Neurodegenerative Activity—In Vitro, In Vivo and Ex Vivo Studies

**DOI:** 10.3390/molecules29184365

**Published:** 2024-09-13

**Authors:** Karolina A. Wojtunik-Kulesza, Monika Rudkowska, Katarzyna Klimek, Jarosław Mołdoch, Monika Agacka-Mołdoch, Barbara Budzyńska, Anna Oniszczuk

**Affiliations:** 1 Department of Inorganic Chemistry, Medical University of Lublin, Chodźki 4a, 20-093 Lublin, Poland; anna.oniszczuk@umlub.pl; 2 Independent Laboratory of Behavioral Studies, Medical University of Lublin, 4A Chodźki, 20-093 Lublin, Poland; monika.rudkowska@umlub.pl (M.R.); barbara.budzynska@umlub.pl (B.B.); 3 Department of Biochemistry and Biotechnology, Medical University of Lublin, 1 Chodźki Street, 20-093 Lublin, Poland; katarzyna.klimek@umlub.pl; 4 Department of Biochemistry and Crop Quality, Institute of Soil Science and Plant Cultivation, State Research Institute, 24-100 Puławy, Poland; jmoldoch@iung.pulawy.pl; 5 Department of Plant Breeding and Biotechnology, Institute of Soil Science and Plant Cultivation, State Research Institute, Czartoryskich 8, 24-100 Puławy, Poland; magacka@iung.pulawy.pl

**Keywords:** *S*-(+)-carvone, neurodegeneration, lipid peroxidation, ROS, passive avoidance, locomotor activity, butyrylcholinesterase, gas chromatography–mass spectrometry

## Abstract

Carvone, a natural monoterpene, has been identified in various plants, giving them a characteristic scent. Enantiomers (*R*-(–) and *S*-(+)) reveal specific biological activities that are successfully used in traditional medicine for their antifungal, antibacterial, antiparasitic, and anti-influenza properties. The presented paper is based on *S*-(+)-carvone, characterized by a specific caraway scent, which revealed rich biological activities both in vitro and in vivo. Thus, the aim of the study was to evaluate the potential anti-neurodegenerative activity of *S*-(+)-carvone, including in vitro experiments (butyrylcholinesterase inhibitory, neuro- and hepatotoxicity as well as neuro- and hepatoprotective activity), in vivo (memory acquisition, locomotor activity), and ex vivo (determination of *S*-(+)-carvone’s level in tissues collected from mice). Results revealed the multidirectional character of *S*-(+)-carvone. It has been shown that *S*-(+)-carvone is capable of butyrylcholinesterase inhibition (40% for 0.025 mg applied onto the plate), and neuroprotection and hepatoprotection at selective concentrations against reactive oxygen species generation and lipid peroxidation along with non-hepatotoxicity character. Additionally, multiple-dose administration of the monoterpene at a dose of 100 mg/kg had a positive influence on memory acquisition. Gas chromatography–mass spectrometry analysis of the plasma and the brain showed that *S*-(+)-carvone can cross the blood–brain barrier and accumulate in the hippocampus (0.217 µg/mg of tissue), a crucial part of the brain associated with cognition and mental functions.

## 1. Introduction

**Figure 1:**
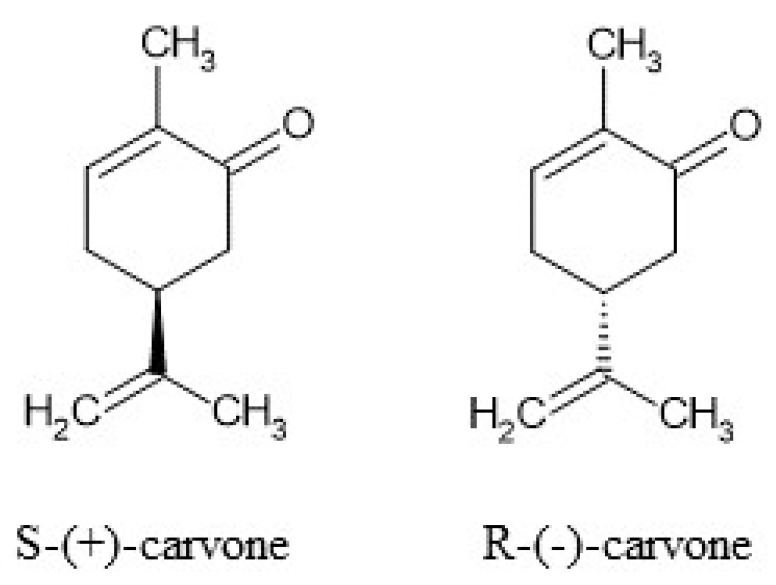
Structures of *S*-(+)-carvone and *R*-(−)-carvone.

Carvone (5-isopropenyl-2-methyl-2-cyclohexanone) is a natural monoterpene (Figure 1) that occurs in the form of two enantiomers: *R*-(−)- and *S*-(+). The enantiomers can be found in various plants, namely spearmint (*Mentha spicata*) and caraway (*Carum carvi* L.) [1]. In accordance with Ali-Shtayeh et al. [2], depending on the season, the amount of carvone in *M. spicata* can increase from 59.09% in winter up to 76.82% in summer. The plants are commonly cultivated and used in the USA, China, and South America (*M. spicata*) as well as in Northern and Central Europe, Siberia, Turkey, and Asia (*Carum carvi* L.). In addition to the aforementioned plants, the monoterpene was identified in *Lippia alba*, *Anethum graveolens* L., and *Mentha cardiac* L. [3].

The biological activity of carvone along with its enantiomers and essential oils (Eos) was evaluated numerous times. It is known that it has several activities, including anti-proliferative, antitumor, anti-hypertensive [4], inhibitory toward proteasome activity, oncogene expressions [5], or anticonvulsive [3] effects. Interesting is also the fact that carvone impacts pain reduction, which was presented by Gonçalves et al. [6]. It was observed that mice treated with 100 and 200 mg/kg of *R*-(−)-carvone revealed a significant decrease in the number of writhes. Additionally, it is known that the analgesic effect is not associated with the opioid system [6].

Carvone activity was also evaluated for anti-neurodegenerative activity. It is known that neurodegenerative disorders constitute one of the most complex groups of disorders strictly associated with dementia. It should be emphasized here that dementia should not be considered a disease because it is rather a syndrome that can be linked to a range of brain disorders [7]. Among the neurodegenerative factors strictly associated with the development of Alzheimer’s disease, the main cause of dementia, are oxidative stress, a disturbance in metal-ion homeostasis, changes in the level of neurotransmitters, accumulation of amyloid-β and neurofibrillary tangles, neuroinflammation, and more [8]. The activity of carvone towards the aforementioned neurodegenerative factors was evaluated both in vitro and in vivo. Available data indicate that the monoterpene can be a good candidate for a multi-directional anti-neurodegenerative agent. The results so far indicate its activity towards free radical scavenging, acetylcholinesterase (AChE) inhibition, and metal-ion reduction/chelation [9,10,11]. It is worth mentioning that in silico studies based on AChE structure revealed that carvone interacts with amino acid residues, creating the enzyme’s active site [10]. Studies performed on rat models showed that the administration of *S*-(*+*)-carvone (10 and 20 mg/kg/bw) induced cerebral I/R induced attenuated neuronal injury, developed antioxidant activity, and decreased glutathione (GSH), acetylcholinesterase and malondialdehyde (MDA) levels [4].

Interesting is also the fact that the enantiomers have a dominant impact not only on the smell of carvone but also on the activity of the monoterpene. It is worth mentioning that the aforementioned anticonvulsive activity was observed in the case of *S-*(+)-carvone, while *R-*(−)-carvone did not exhibit this property. Similar differences were observed in other studies: *S-*(+)-carvone caused an increase in systolic and diastolic blood pressure without emotional measures whereas *R-*(−)-carvone led to increases in pulse rate and diastolic blood pressure along with subjective restlessness [12].

The presented paper aimed to supplement knowledge on the topic of *S*-(+)-carvone activity towards butyrylcholinesterase inhibition in vitro, hepatotoxicity and hepatoprotective activity against ROS generation, and lipid peroxidation based on cell lines, memory acquisition, and locomotor activity in a mice model as well as determination of the level of carvone in the plasma and brain collected from mice using gas chromatography–mass spectrometry (GC-MS).

## 2. Results

### 2.1. In Vitro Study Results

#### 2.1.1. BChE Inhibitory Activity

The presented Marston method is one of the first steps toward the determination of acetylcholinesterase/butyrylcholinesterase (AChE/BChE) inhibitory activity in the examined compounds. The obtained results can be considered as the basis for future more detailed analyses of enzyme inhibitory activity using more advanced techniques. The assay was performed for four amounts of the compounds, namely 0.05 mg, 0.025 mg, 0.01 mg, and 0.001 mg. The selection of the amounts was based on previously conducted experiments. The ethanol used as a solvent was tested for AChE inhibition. The tests showed no significant effect on enzyme activity.

The results were analyzed using the Sorbfil TLC Videodensitometer (ver. 2.0) program, which allowed us to assess the activity of the compounds based on changes in the color of the spots with the applied compound compared to the background of the plate that does not contain active compounds. It should be emphasized here that the analysis with the software allows us to avoid the negative impact of spot enlargement resulting from the high volatility of the sample. Additionally, the comparative analysis allowed the obtained results to be compared to the activity of the reference substance, in this case, galantamine. Results obtained with the use of Sorbfil software are presented in Table 1.

The obtained results revealed the activity of *S*-(+)-carvone towards BChE, which was only 40% in relation to galantamine, which was assumed to be 100%. It is worth mentioning that the activity was almost equal for 0.05 mg and 0.025 mg; whereas, lower amounts (0.01 mg and 0.001 mg) significantly decreased inhibitory activity.

#### 2.1.2. Neurotoxicity and Hepatotoxicity of *S*-(+)-Carvone

**Figure 2:**
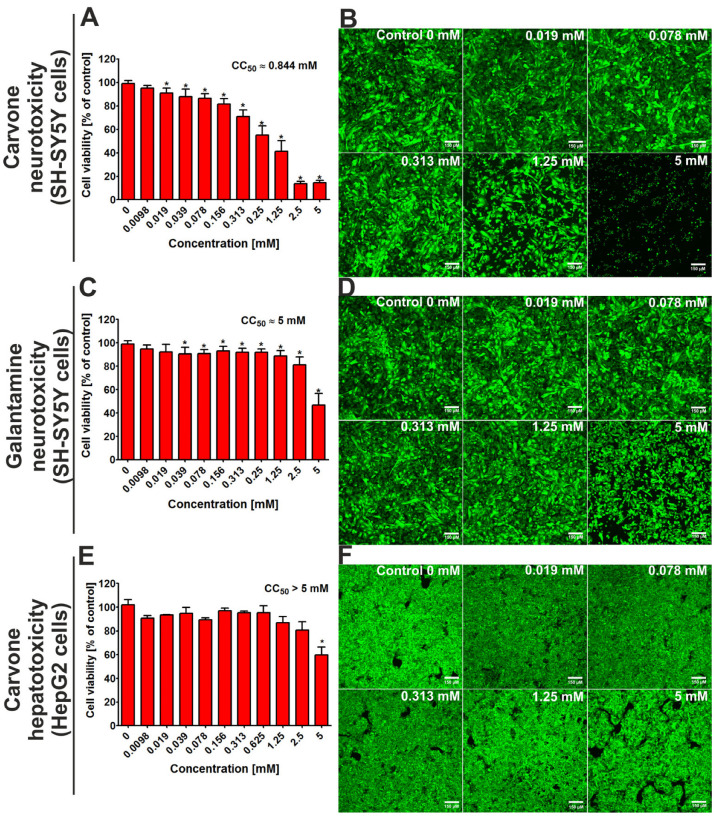
The viability of the SH-SY5Y cell line (human neuroblastoma cells) and the HepG2 cell line (human hepatocellular as well as carcinoma cells) after 48-h incubation with *S*-(+)-carvone. Galantamine was evaluated as a reference drug. In the case of hepatotoxicity, the results for galantamine were presented in detail in our previous work [13]. Quantitative evaluation of cytotoxicity was assessed using the MTT assay (**A**,**C**,**E**); * Statistically significant differences compared to control cells (incubated without tested compound—marked as Control 0 mM), Student *t*-test, *p* < 0.05. Visualization of live (green fluorescence) and dead cells (red fluorescence) after staining with Live/Dead Double kit (**B**,**D**,**F**); magnification 100×, scale bar = 150 μM.

After 48 h of incubation, the MTT assay indicated that the CC_50_ value (cytotoxic concentration that reduced 50% of cell viability) for *S*-(+)-carvone against SH-SY5Y cells was close to 0.844 mM (Figure 2A). At the same time, the CC_50_ value for galantamine (the reference drug) was close to 5 mM. Confocal microscope analysis (Figure 2B,D) confirms the results obtained from the MTT assay and demonstrates that *S*-(+)-carvone was more cytotoxic toward neuronal cells when compared to galantamine. In turn, the evaluation of hepatotoxicity showed that the CC_50_ value for carvone was above 5 mM (Figure 2F), while for galantamine, it was close to 5 mM [13].

#### 2.1.3. Neuroprotective and Hepatoprotective Activity of *S*-(+)-Carvone against Oxidative Stress and Lipid Peroxidation

**Figure 3:**
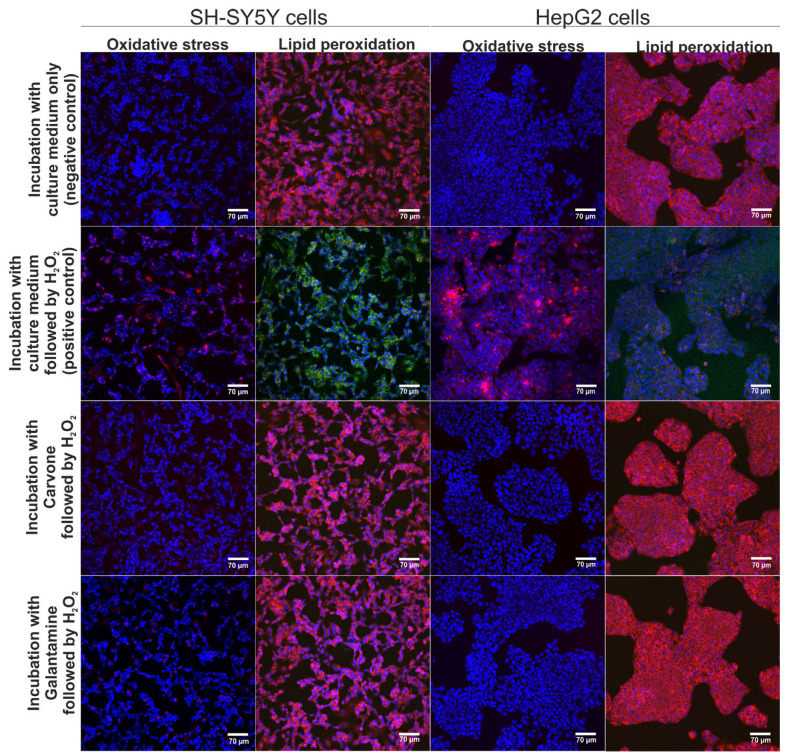
The CLSM images demonstrate the neuroprotective and hepatoprotective activity of *S*-(+)-carvone after induction of oxidative stress and lipid peroxidation. Additionally, galantamine was used as a reference drug. Cells were stained with Hoechst 33342 for nuclei visualization (blue fluorescence) and with CellROX^TM^ Deep Red Reagent for visualization of oxidative stress (red fluorescence) or with Image-IT^TM^ Lipid Peroxidation for visualization of lipid peroxidation (green fluorescence) or lack of lipid peroxidation (red fluorescence); magnification 200×, scale bar = 70 μm.

The neuroprotective and hepatoprotective properties of *S*-(+)-carvone were assessed via CLSM observations (Figure 3). According to the results obtained within the MTT assay (previous experiment), *S*-(+)-carvone at concentrations of 0.078 mM (for SH-SY5Y cells) and 0.625 mM (for HepG2 cells) were utilized. These concentrations were selected as they did not cause a reduction of cell viability below 90%. It was observed that pre-treatment with *S*-(+)-carvone or galantamine (reference drug) significantly decreased the generation of ROS in SH-SY5Y and also HepG2 cells subsequently incubated with H_2_O_2_, which as a consequence, led to the inhibition of oxidative stress and lipid peroxidation in cells after treatment with H_2_O_2_ (Figure 3). On the other hand, the H_2_O_2_ exhibited high oxidative potential to stimulate oxidative stress and lipid peroxidation in SH-SY5Y and HepG2 cells maintained only in the culture medium (positive control). Thus, these results demonstrated that *S*-(+)-carvone compared to the standard drug galantamine had a strong neuroprotective and hepatoprotective potential in vitro.

### 2.2. In Vivo Assays Results

#### 2.2.1. The Effect of Single-Dose and Multiple-Dose Administration of S-(+)-Carvone on Memory Acquisition Processes in the Passive Avoidance (PA) Test

**Figure 4:**
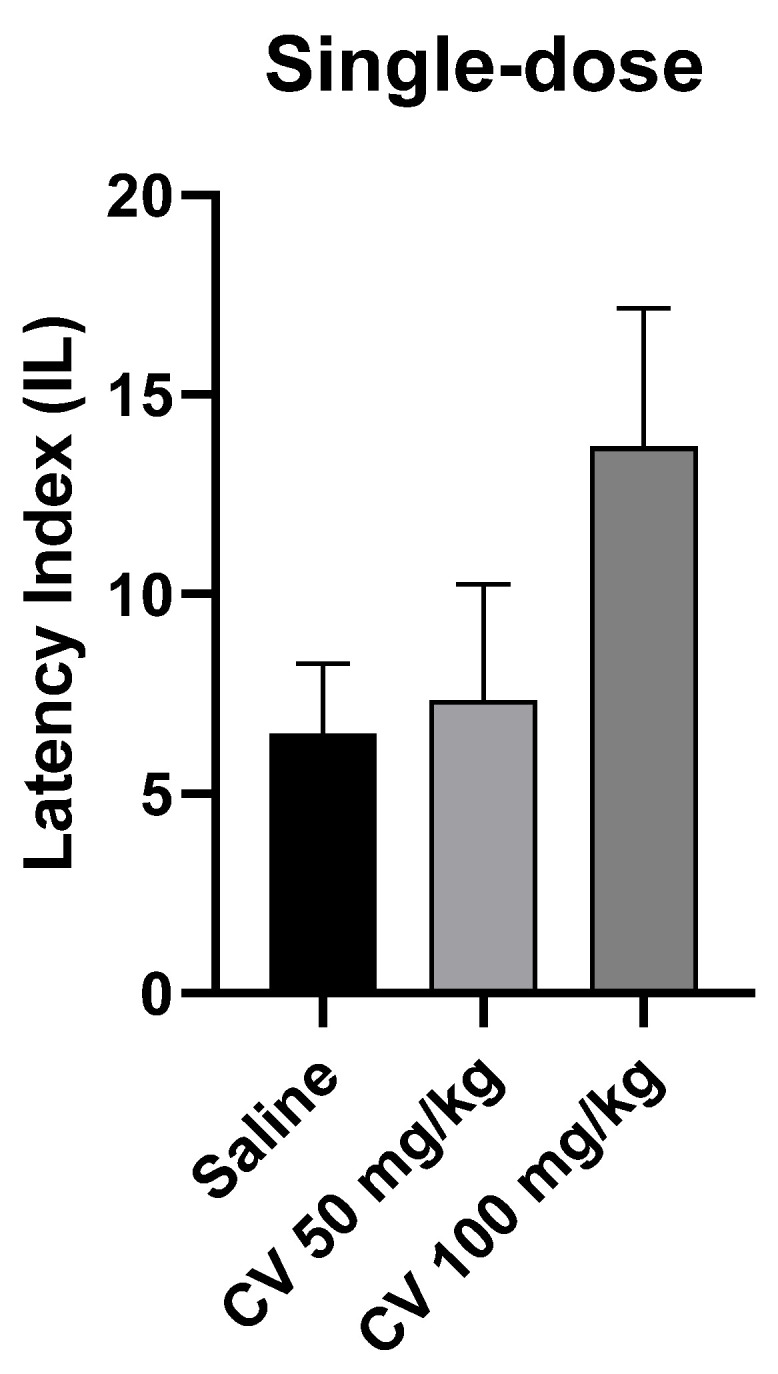
The effect of single-dose administration of *S*-(+)-carvone (CV) on memory acquisition processes in the PA test in mice. CV (50 or 100 mg/kg i.p.) or saline solution (i.p.) were administered 30 min before training. Subsequently, 24 h after training, the animals were re-tested. The results were expressed as a latency index (IL) and presented as a mean ± SEM, *n* = 10. The statistical analysis of the data was performed using a one-way analysis of variance (ANOVA) followed by the post hoc Dunnett’s test.

A one-way ANOVA test showed that single-dose administration of *S*-(+)-carvone did not affect the long-term memory acquisition processes in the PA test in mice [F (2, 27) = 1.972; *p* = 0.1588]. Dunn’s post hoc test confirmed that *S*-(+)-carvone at doses of 50 mg/kg and 100 mg/kg did not significantly affect the memory processes related to acquisition. A higher dose of *S*-(+)-carvone (150 mg/kg) significantly reduced the IL value compared to the saline-treated control group (*p* < 0.01) (Figure 4).

**Figure 5:**
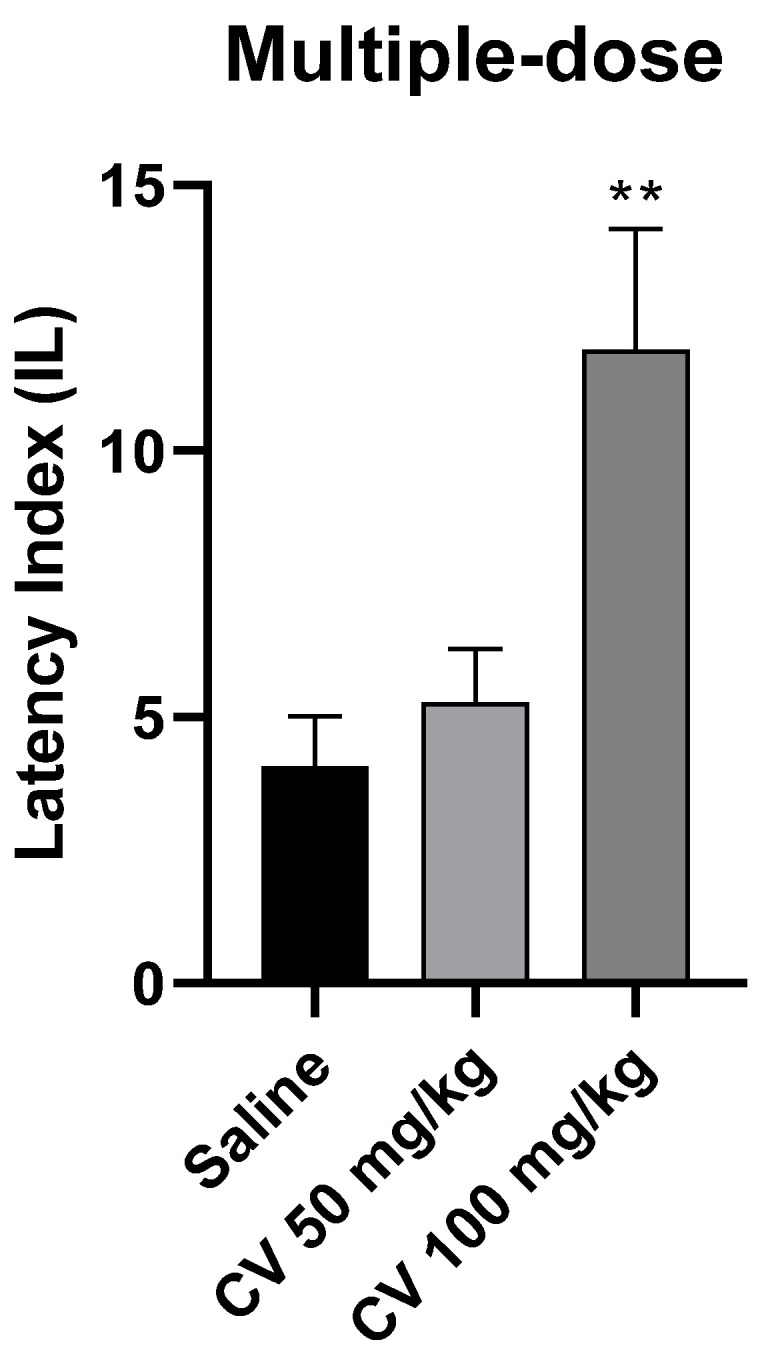
The effect of multiple-dose administration of *S*-(+)-carvone (CV) on the memory acquisition processes in the PA test in mice. CV (50 and 100 mg/kg, i.p.) or saline solution (i.p.) were administered for 6 days, twice daily. On the seventh day, the appropriate group of mice received CV (50 or 100 mg/kg, i.p.) or saline solution (i.p.) 30 min before training. Subsequently, 24 h after training, the animals were re-tested. The results were expressed as a latency index (IL) and presented as a mean ± SEM, *n* = 9–10. The statistical analysis of the data was performed using a one-way analysis of variance (ANOVA) followed by the post hoc Dunnett’s test. ** *p* < 0.01 vs. saline-treated control group.

A one-way ANOVA analysis showed that multiple-dose administration of *S*-(+)-carvone affects the processes of acquisition of long-term memory in the PA test in mice [F (2, 26) = 7.331; *p* = 0.003]. According to Dunnett’s post hoc test, *S*-(+)-carvone at a dose of 50 mg/kg did not significantly affect the memory processes related to acquisition. In turn, *S*-(+)-carvone at a dose of 100 mg/kg significantly increased the IL value compared to the saline-treated control group (*p* < 0.01) (Figure 5).

#### 2.2.2. The Effect of Single-Dose and Multiple-Dose Administration of S-(+)-Carvone on Memory Acquisition Processes Impaired by a Single-Dose Injection of Scopolamine in the PA Test

**Figure 6:**
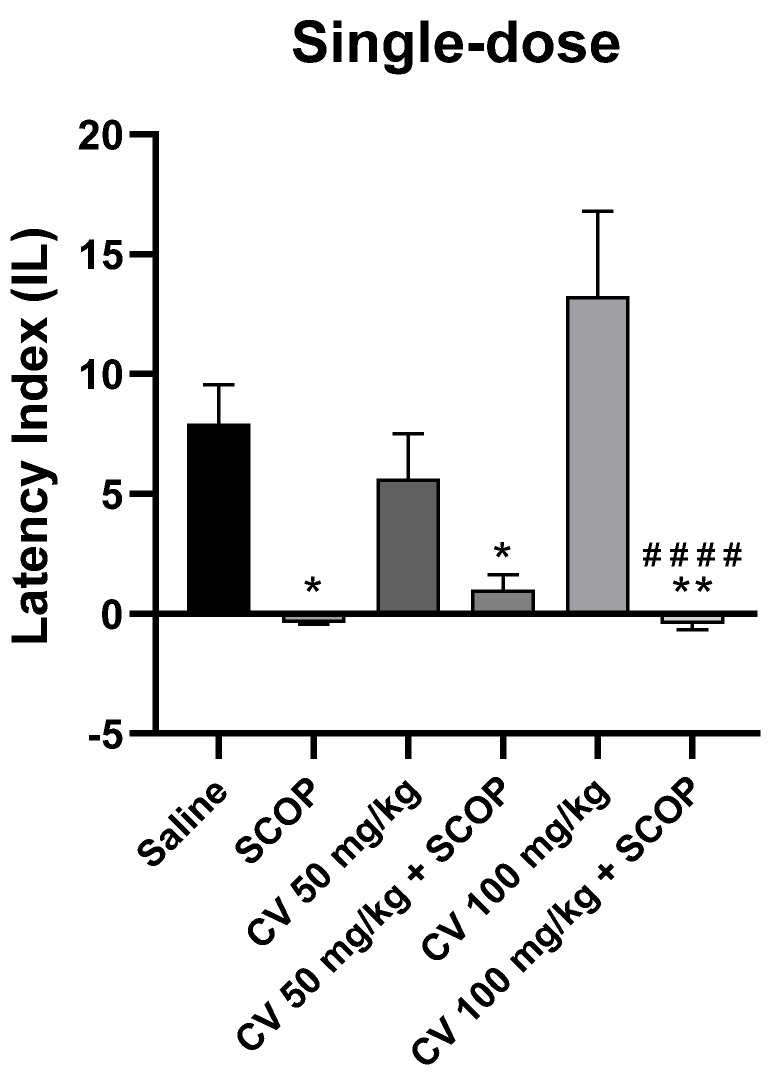
The effect of single-dose *S*-(+)-carvone (CV) administration on memory acquisition processes is impaired by a single-dose injection of scopolamine in the PA test. CV (50 or 100 mg/kg, i.p.), saline solution (i.p.), or scopolamine (1 mg/kg, s.c.) were administered 30 min before training. Subsequently, 24 h after training, the animals were re-tested again. The results were expressed as a latency index (IL) and presented as a mean ± SEM, *n* = 8–10. The statistical analysis of the data was performed using a two-way analysis of variance (ANOVA) followed by the post hoc Bonferroni’s test. * *p* < 0.05; ** *p* < 0.01 vs. saline-treated control group; #### *p* < 0.0001 vs. CV 100 mg/kg.

After single-dose administration, a two-way analysis of variance (ANOVA) showed statistically significant changes in scopolamine pretreatment [F (1, 48) = 42.52; *p* < 0.0001] and *S*-(+)-carvone treatment [F (2, 48) = 1.949; *p* = 0.1535]. Interactions between the scopolamine and *S*-(+)-carvone were observed [F (2, 48) = 3.611; *p* = 0.0346]. *S*-(+)-carvone, at a dose of 150 mg/kg, significantly impaired memory processes in the PA test and was excluded from further analysis (Figure 6).

The Bonferroni’s post hoc test showed a decrease in IL values following the use of scopolamine (1 mg/kg) [*p* < 0.05] and after the single-dose CV treatment (50 or 100 mg/kg) with scopolamine compared to the control group [*p* < 0.05 or *p* < 0.01, respectively]. No statistically significant differences were found between the group receiving scopolamine alone (1 mg/kg) and the groups receiving the combination of scopolamine with CV (50 or 100 mg/kg) [*p* > 0.9999]. This suggests that CV at these doses does not eliminate the adverse effects of scopolamine on memory processes. *S*-(+)-carvone at a dose of 100 mg/kg increased the IL, but the result did not reach the level of statistical significance compared to the control group. However, significant differences in the effect on memory were observed between the groups treated with CV (100 mg/kg) and CV (100 mg/kg) with scopolamine [*p* < 0.0001].

After short-term administration, a two-way ANOVA showed statistically significant changes in scopolamine pretreatment [F (1, 50) = 30.85; *p* < 0.0001] and in *S*-(+)-carvone treatment [F (2, 50) = 7.443; *p* = 0.0015], while interactions between scopolamine and *S*-(+)-carvone were observed [F (2, 50) = 5.386; *p* = 0.0076].

**Figure 7:**
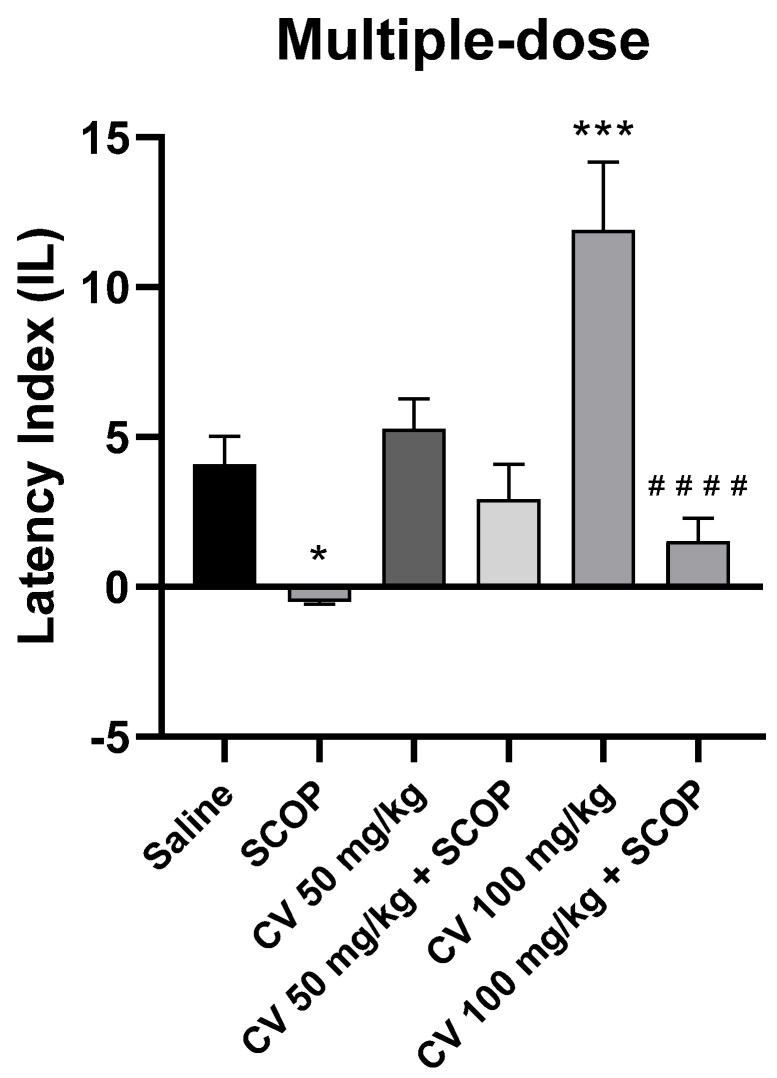
The effect of multiple-dose *S*-(+)-carvone (CV) administration on memory acquisition processes is impaired by a single-dose injection of scopolamine in the PA test. CV (50 or 100 mg/kg, i.p.) or saline solution (i.p.) was administered for 6 days, twice daily. On the seventh day, the appropriate group of mice received CV (50 or 100 mg/kg, i.p.) or saline solution (i.p.) or scopolamine (1 mg/kg, s.c.) 30 min before training. The animals were tested again 24 h after training. The results were expressed as a latency index (IL) and presented as a mean ± SEM, *n* = 9–10. The statistical analysis of the data was performed using a two-way analysis of variance (ANOVA) followed by the post hoc Bonferroni’s test. * *p* < 0.05; *** *p* < 0.001 vs. saline-treated control group; #### *p* < 0.0001 vs. CV 100 mg/kg.

The post-hoc Bonferroni’s test confirmed that scopolamine (1 mg/kg) treatment impaired the acquisition of memory [*p* < 0.05]. There were no statistically significant differences between the group administered scopolamine alone (1 mg/kg) and the groups treated with both scopolamine and CV (50 or 100 mg/kg) [*p* > 0.05]. An improvement in memory acquisition induced by *S*-(+)-carvone at a dose of 100 mg/kg was observed [*p* < 0.001]. Furthermore, a significant difference was found in IL values between the group treated with *S*-(+)-carvone (100 mg/kg) and the group receiving a combination of *S*-(+)-carvone (100 mg/kg) with scopolamine [*p* < 0.0001] (Figure 7).

#### 2.2.3. The Effect of S-(+)-Carvone on Locomotor Activity in Mice

Regarding the effect of S-(+)-carvone on the locomotor activity of mice, a one-way ANOVA statistical analysis showed that S-(+)-carvone, both after single-dose and multiple-dose administration, significantly impaired locomotor activity compared to the control group within 30 min of administration of the compound ([F (2, 19) = 54.09; *p* = <0.0001]; [F (2, 21) = 9.564; *p* = 0.0011], respectively). Similarly, single-dose and multiple-dose administration of S-(+)-carvone reduced the distance traveled by animals over 60 min ([F (2, 19) = 27.15; *p* < 0.0001]; [F (2, 21) = 7.165; *p* = 0.0042], respectively). Dunnett’s post hoc test confirmed impaired locomotor activity after S-(+)-carvone treatment of mice. Detailed data are provided in Table 2.

### 2.3. Ex Vivo Assay Results

#### GC-MS Analysis of S-(+)-Carvone in Biological Material

GC-MS analysis of S-(+)-carvone was performed in tissues (plasma, hippocampus, and remnants of the brain) collected from mice used in in vivo experiments after multiple-dose administration of monoterpene at 100 mg/kg. Taking into account the fact that single-dose administration of S-(+)-carvone did not influence memory acquisition, the GC-MS for the issues was not performed.

Analysis revealed that S-(+)-carvone is present in all tissues after multiple-dose administration (Table 3).

It should be emphasized that *S*-(+)-carvone was identified in all study tissues after multiple-dose administration of the terpene. Significant is the fact that it was detected in the whole brain as well as the hippocampus which is crucial in neurodegenerative changes associated with memory and cognitive functions. It must be underlined that the monoterpene was accumulated in this crucial part of the brain (0.217 µg/mg in comparison to the remnant of the brain 0.045 µg/mg). This fact also indicates the ability of *S*-(+)-carvone to cross the blood–brain barrier.

The chromatogram from the analysis is placed in the Supplementary Material.

## 3. Discussion

Carvone, commonly known as monoterpene, was evaluated for various biological activities. Among them are in vitro and in silico studies used as the basis for in vivo and ex vivo assays. In vitro evaluation allows us to determine the further direction of research.

The first part of the studies presented in the paper was based on BChE inhibitory activity with the use of the Marston assay. The obtained results revealed that *S*-(+)-carvone is able to inhibit the enzyme. The activity was explicit for higher amounts of monoterpene applied onto the TLC plate (0.05 mg and 0.025 mg), which in reference to galantamine, constitutes 38% and 40% of its activity, respectively. Similar studies based on AChE inhibition activity were presented in a previous paper [10]. There is a limited number of studies based on *S*-(+)-carvone—AChE/BChE inhibitory assay; however, activity can be inferred indirectly by examining the activity of the essential oils that are rich in this monoterpene. An example can be *M. spicata*, which revealed AChE IC_50_ and BChE IC_50_ for EO equal to 78.3 µg/mL and 192.1 µg/mL, respectively [14]. Similarly, studies were performed by Orhan et al. who presented carvone BChE inhibitory activity equal to 62.4% [15]. The preliminary studies towards AChE and BChE activity are important when considering anti-neurodegenerative activity because disturbances in the amount of neurotransmitters are presented as one of the main causes or consequences of changes occurring in the brain.

An important point when considering active substances and their potential use is to check their neurotoxicity and hepatotoxicity. According to Kuete and Efferth [16], the terpenes should be classified as follows: strong cytotoxic (CC50 < 0.1 mM), moderate cytotoxic (>0.1 mM–<0.3 mM), low cytotoxic (>0.3 mM–<1 mM), and no cytotoxic (>1 mM). In vitro studies performed on SH-SY5Y and HepG2 cells revealed that *S*-(+)-carvone is a safe compound (CC_50_ is approx. 0.844 mM and > 5 mM, respectively). It means that it should be classified as a low neurotoxic or no hepatotoxic compound. Additionally, the high inhibition of ROS generation and lipid peroxidation allows us to assume that *S*-(+)-carvone reveals strong neuroprotective and hepatoprotective activity in vitro. Considering the fact that most neurodegeneration disorders are strictly associated with oxidative stress, the antioxidant activity of *S*-(+)-carvone is a significant advantage. The studies performed on cell lines, which were performed using other assays, confirmed the antioxidant activity of *S*-(+)-carvone [11]. Oxidative processes are responsible for various harmful changes in organisms leading to carcinoma, neurodegeneration, and more. The determination of free radical scavenging activity as well as antioxidant activity of the analyzed compound is a first step towards its detailed characterization. The neuroprotective and hepatoprotective activity against ROS generation and lipid peroxidation of *S*-(+)-carvone are an important part of the research that is part of the detailed characterization of the compound towards potential anti-neurodegenerative properties. It is worth underlining, that our previous study [13] demonstrated that the values of CC_50_ for galantamine (reference drug) and another monoterpene, citral, against HepG2 cells were close to approx. 5 mM and to approx. 0.57 mM, respectively. Thus, the current experiment showed that *S*-(+)-carvone possessed a lower hepatoxic effect not only compared to citral but also to galantamine.

Hence, it should be justified to perform the next step in the future, which will include the characterization of *S*-(+)-carvone activity performed using antioxidant enzymes in in vivo models including antioxidant enzymes.

One of the most popular and often used approaches in behavioral studies is the impairment of memory by a specific substance such as scopolamine. It is known that scopolamine, a blocker of muscarinic cholinergic receptors (mAChRs), causes disturbances in both short-term and long-term memory [17]. The drug administration influences the reduction of ACh levels in mice as well as oxidative stress and changes in the metabolism of selected antioxidants including glutathione which indirectly leads to memory disturbance [18]. Considering the information, it can be assumed that drugs able to reverse the effect of scopolamine are able to act by AChE inhibition or significant impact on the reduction of oxidative stress.

Detailed analysis of passive avoidance test results showed that *S*-(+)-carvone at a dose of 50 mg/kg and 100 mg/kg did not significantly affect memory processes related to the acquisition in the case of single-dose administration. In the case of multiple-dose administration, the impact on the processes of acquisition of long-term memory was observed. Simultaneously, the monoterpene did not eliminate the adverse effect of scopolamine on memory processes. It should be underlined that interactions between scopolamine and *S*-(+)-carvone were observed and a significant difference was found in IL values between the group treated with *S*-(+)-carvone (100 mg/kg) and the group receiving a combination of *S*-(+)-carvone (100 mg/kg) with scopolamine [*p* < 0.0001] (Figure 6 and Figure 7). The studies did not indicate strong activity of *S*-(+)-carvone towards AChE inhibition or antioxidant activity at the presented conditions. The fact that *S*-(+)-carvone did not reverse the effects of scopolamine indicates that it works in a different mechanism than those indicated above. Another possible scenario is that the effect of scopolamine is so strong that *S*-(+)-carvone, despite its inhibitory and antioxidant properties demonstrated in in vitro tests [10,11], is not strong enough to reverse its effect. At this point, it is significant to mention that the affinity of scopolamine for muscarinic receptors is <1 nM and is similar to ACh. However, a significant fact is that *S*-(+)-carvone impacted memory acquisition. Interactions between *S*-(+)-carvone and scopolamine were also observed. It can suggest that *S*-(+)-carvone is able to impact memory in the cholinergic mechanism along with others, such as antioxidants, which are not as strong as the scopolamine effects.

A significant side effect of *S*-(+)-carvone administration was the impairment of the locomotor activity of mice observed both after single-dose and multiple-dose administration. It should be emphasized that despite the negative influence of *S*-(+)-carvone on locomotor activity, the impact on memory processes remained unchanged. A similar effect, observed as a decrease in mice activity, was described by Buchbauer et al. [19]. Other studies were based on a comparison of the effect of carvone enantiomers on the human autonomic nervous system [20]. In this case, no significant effect of *S*-(+)-carvone on subjective calmness was found.

Significant study results were obtained for GC-MS analysis based on tissues collected from mice subjected to experiments. The analysis included tissues collected from mice after multiple-dose administration of *S*-(+)-carvone due to single-dose administration did not bring significant results in behavioral studies. The analysis included the plasma, hippocampus, and remnants of the brain. The administered monoterpene was identified in all tissues and importantly, in the case of the brain, the monoterpene accumulated in the hippocampus. It is known that this part of the brain is pivotal in cognitive function and social behavior; hence, it is desirable that the drugs reach there [21]. Detection of the presence of carvone in the brain confirms its ability to cross the blood–brain barrier (BBB), which is crucial for potential anti-neurodegenerative agents. Various results obtained in the mice model indicate the ability of carvone to access BBB [22]. At this point, it should be emphasized that the determination of carvone’s presence in biological tissues is difficult due to the high volatility of the compound, and the availability of research results in the literature is limited. An ADMET analysis of the carvone molecule allows us to predict that it is a promising compound. Additionally, the monoterpene molecule satisfies Lipinski’s rule of five [22,23]. The obtained results revealed activity in the PA test after multiple-dose administration but the simultaneous effect of scopolamine, a blocker of muscarinic cholinergic receptors, was not reversed [17]. This fact indicates that carvone activity results from other mechanisms of action that can be associated with one of those presented previously.

As mentioned previously, *S*-(+)-carvone was evaluated for its anti-neurodegenerative activity, such as antioxidant properties, AChE inhibitory properties, and metal-ion reduction/chelation [9,10,11]. It is worth mentioning that the compound revealed good activity toward most of the analyzed neurodegenerative factors. In the case of free radical scavenging (DPPH assay), during the first minutes of the study, the monoterpene scavenged almost 80% of the free radicals [11]. Similar high activity was observed for AChE inhibition in Ellman’s and Marston’s assays as well as the molecular docking simulation [10]. Another important activity of *S*-(+)-carvone was observed in FRAP, CUPRAC, and the ferrozine-based chelation assay [24,25]. The multi-directional activity was the basis for more advanced studies towards anti-neurodegenerative factors as well as the ability of *S*-(+)-carvone to counteract dementia symptoms.

Considering the fact that the brain is the command center of our body, any damage causes changes in our body. Apart from disturbances in speech, movement, stability, and balance, there are significant changes in mood and behavior such as anxiety, depression, apathy, agitation, or space disturbances [26]. In the context of neurodegenerative diseases, carvone has been tested for depression, sedation, nociception, seizure, and local anesthesia [1,27,28,29]. The activities were evaluated with the use of various models such as Swiss mice, Wistar rats, or frog’s sciatic nerve. Obtained results revealed that *S*-(+)-carvone (100 mg/kg) increases pentobarbital sleeping duration whereas a higher dose (200 mg/kg) improved the latency of convulsions produced by PTZ and PIC. In relation to neurodegeneration, an important aspect is depression. Both enantiomers of carvone revealed depressive effects at high doses of monoterpene (LD50 = 400–500 mg/kg) [27]. An important fact that should be considered during behavioral studies is the potential anesthetic activity of the studied compound. In accordance with Faliagkas et al. [28], carvone reveals activity at concentrations of 10 and 20 mM. An analysis of the results revealed that both enantiomers act in the same way as 10 mM of lidocaine, a reference substance, but carvone was 3–4 times less active in terms of reaction time [30].

A detailed analysis of carvone as a potential anti-neurodegenerative agent revealed that, in addition to the aforementioned activities, the compound has significant interactions with γ-aminobutyric acid (GABA). It is known that the major inhibitory neurotransmitter in the vertebrate and invertebrate nervous system is the target of many studies in the field of neurodegeneration and dementia [31]. An analysis of the inhibitory activity of carvone towards GABA suggests that both carvone isomers can inhibit GABA-induced stimulation of [3H]flunitrazepam binding, which indicates their interaction with GABAA-R as negative allosteric modulators [1]. Based on considerations of Brosnan et al. [32], carvone, due to molar water solubility (7.8 mM) higher than the cutoff values for GABAA, should exhibit the ability to modulate the functions of the ion channel.

Significant studies based on the influence of carvone on memory in mice were conducted by Laserte-Cia et al. [33]. Scientists decided to evaluate the activity using inhalation procedures as an interesting way to administer volatile compounds to organisms and evaluate their activity on the CNS. The experiment based on BALC/c and C57BL/6J mice revealed important carvone activity, namely inhalation improved memory capacity in the first group of mice; whereas, in the case of C57BL/6J, the observed impairment was the same capacity. The interpretation of the results is based on the fact that cytokines due to learning and memory processes are strictly associated with cytokines production by immune cells [34]. It is known that IL-1β, IL-6, TNF-α IFN-γ, IL-10, IL-4, and BDNF have a significant influence on synaptic plasticity and changes in hippocampal-dependent learning and memory tests. An analysis of the obtained studies revealed that carvone caused a significant increase in IL-1β, IL-6, and TNF-α in BALC/c mice; whereas, a reduction of IL-1β and an increase in IFN-γ were observed in C57BL/6 mice [34]. It is significant to underline that carvone can enter the CNS by nasal epithelium, bypassing the BBB, and have a direct pharmacological effect through the olfactory receptors expressed in neurons [35]. It should be underlined that the monoterpene, depending on dosage, can involve side effects such as depression which must be particularly taken into account when selecting a dose that is to have a positive effect on brain functioning. In the future, detailed analysis should be provided.

## 4. Materials and Methods

### 4.1. Materials

The following reagents were purchased from Sigma Aldrich (St. Louis, MO, USA): *S*-(+)-carvone (>96%), butyrylcholinesterase, type V-S from Electrophorus electricus, albumin from bovine serum, 1-naphthyl acetate, Tween 20, Trizma^®^ (2-amino-2-(hydroxymethyl)-1,3-propanediol) hydrochloride solution (1M, pH 7.8), acetylthiocholine iodide (≥99%), DTNB (5,5’-dithiobis(2-nitrobenzoic acid)), galantamine hydrobromide from Lycoris sp. (>94%) and Fast Blue B salt 95%. Solvent analytical purity grades were obtained from Polish Reagents (Gliwice, Poland). Additionally, thin layer chromatography plates (HPTLC silica gel 60) (Merck, Germany) were used. Human hepatocellular carcinoma cells (HepG2 cell line, HB-8065) and Eagle’s Minimum Essential Medium (EMEM) were supplied by American Type Culture Collection (ATCC), London, UK. Penicillin/streptomycin solution, Live/Dead Cell Double Staining Kit, and MTT were purchased from Merck (St. Louis, MO, USA), while fetal bovine serum (FBS) and CellROX^TM^ Deep Red Reagent as well as Image-IT^TM^ Lipid Peroxidation Kit were purchased from Pan-Biotech (Aidenbach, Germany) and Invitrogen, ThermoFisher Scientific (755 US-202, Branchburg, NJ 08876, USA), respectively.

### 4.2. Methods

#### 4.2.1. In Vitro Assays

##### Butyrylcholinesterase (BChE) Inhibitory Assay on TLC Plates According to the Marston Method

The assay was performed based on the method presented by Marston et al. [36] using chromatographic HPTLC silica gel 60 (F254) plates. First of all, the TLC plate was eluted with ethanol and activated for 40 min at 105 °C. The next step was terpene application onto the plate using an automatic applicator, Desaga AS-30 (Biostep GmbH, Burkhardtsdorf, Germany). In order to reduce the high volatility of *S*-(+)-carvone, the compound was diluted in ethanol. The following amount of *S*-(+)-carvone was applied [mg]: 0.05; 0.025; 0.01 and 0.001. The amount of the applied compound was decided on the basis of previously conducted tests for acetylcholinesterase [10]. Galantamine was used as a reference standard. The solution of the reference standard (1 mg/mL in ethanol) was applied to the plates in the same amount as the investigated terpene. The next step of the assay was performed in accordance with [10]. The obtained results were analyzed using the Sorbfil TLC Videodensitometer program (v. 2.0).

##### Assessment of Neurotoxicity and Hepatotoxicity In Vitro

This experiment was performed as previously described in detail [13]. Briefly, SH-SY5Y or HepG2 cells were seeded on 96-well plates and incubated for 24 h for their attachment. Then, the culture medium was discarded and the serial dilutions (0.0098–5 mM) of *S*-(+)-carvone were added. Moreover, galantamine (as a reference drug) was applied. The MTT assay was performed in order to assess the cell viability after 48 h of incubation. Moreover, the cells were stained with a Live/Dead Cell Double Staining Kit and visualized under a confocal laser scanning microscope (CLSM).

##### Determination of Oxidative Stress and Lipid Peroxidation

This experiment was performed as previously described in detail [13]. In brief, cells were prepared and treated according to the procedure described in the Section Assessment of neurotoxicity and hepatotoxicity in vitro, but using *S*-(+)-carvone or galantamine at a concentration of 0.078 mM (for SH-SY5Y cells) or 0.625 mM |(HepG2 cells). After 48 h, oxidative stress was generated by a 1.5-h treatment with 500 μM H_2_O_2_. The negative control of the experiment was cells incubated with only a culture medium for 49 h, while the positive control was cells incubated with a culture medium for 48 h, followed by a 1.5-h treatment with 500 μM H_2_O_2_. Oxidative stress and lipid peroxidation were evaluated using CellROX^TM^ Deep Red Reagent or Image-IT^TM^ Lipid Peroxidation Kit (both from Invitrogen, ThermoFisher Scientific). Additionally, Hoechst 33342 dye (Merck) was used for cell nuclei staining. The cells were visualized under CLSM.

#### 4.2.2. In Vivo Assays

##### Animals

The investigation involved native adult male Swiss mice sourced from the Centre of Experimental Medicine at the Medical University of Lublin. These mice, initially weighing between 20 and 25 g, underwent testing sessions conducted between 8:30 a.m. and 4:00 p.m. They were accommodated in colony cages within a controlled laboratory environment, featuring a natural 12/12-h light cycle, a constant temperature set at 21 ± 1 °C, air humidity maintained within the range of 50 ± 5%, and air exchange transpiring at a rate of 15/h. Moreover, they were provided with unrestricted access to tap water and food (Altromin, AnimaLab, Im Seelenkamp 20, 32791 Lage, Germany). Following a 7-day acclimatization period, the mice were randomly distributed into experimental groups, each comprising 8–10 individuals.

##### Ethical Declaration

All research was conducted following the ARRIVE guidelines and the European Community Council Directive for the Care and Use of Laboratory Animals of 22 September 2010 (2010/63/EU) and approved by the Local Ethics Committee in Lublin, Poland (Permission No: 42/2022).

##### Drugs

Scopolamine hydrobromide and *S*-(+)-carvone were used in the experiment. Scopolamine was dissolved in a saline solution of 0.9% NaCl and administered subcutaneously (s.c.). *S-*(+)-carvone was suspended in 1% Tween 20 solution and administered intraperitoneally (i.p.). All substances were administered in a volume of 10 mL/kg 30 min before training in memory tests or immediately before assessing locomotor activity. Fresh drug solutions were prepared every day before administration. Control groups received saline injections at the same time and in the same volume as their corresponding medications. The doses of *S*-(+)-carvone and scopolamine were determined based on previous studies [6,15,16,32].

##### Experimental Procedure and Treatment

Passive Avoidance Task

The passive avoidance (PA) task is predicated upon the innate aversion of rodents to brightly illuminated environments and serves as a metric for assessing long-term memory [37]. By modulating the timing of drug administration, the PA test allows for the investigation of distinct memory phases within various testing protocols. In our investigation, pharmacological agents were administered prior to the training session, thereby impeding the process of information acquisition.

The experimental apparatus consisted of an acrylic box partitioned by sliding doors into two discrete compartments: one illuminated and the other maintained in darkness. The flooring of the apparatus comprised stainless steel bars utilized as conduits for the delivery of electric stimuli.

On the first day of trials, following drug administration, the mice were introduced into the illuminated section of the box with the door closed, allowing for 30 s of free exploration. After this period, the sliding doors were raised, granting access to the darkened compartment. When the mouse entered the dark chamber, signified by contact of all four paws, the sliding doors were promptly closed, and a mild electric current (2 s duration, 0.2 mA intensity) was delivered through the floor. The latency period for entry into the dark compartment was recorded as TL1, with observations extending for a duration of 300 s. In cases where the animal did not move to the darkened part of the apparatus within this time, it was manually placed into the darkened chamber, the door was closed, and a mild electrical stimulus was delivered through the floor. In such occurrences, TL1 was recorded as 300 s.

Subsequent to a 24-h interval, the same mice were individually reintroduced into the illuminated section of the apparatus with the sliding doors closed. Following a 30-s adaptation phase, the partition between compartments was lifted, and the time taken by the mice to enter the dark chamber was recorded as TL2. No electric stimulus was administered during this trial. In instances where the subject failed to enter the darkened section within 300 s, the trial was terminated, and TL2 was registered as 300 s [38].

Locomotor Activity

The locomotor activity of the mice was evaluated using an Opto-Varimex-4 Auto-Track actimeter (Columbus Instruments, Columbus, OH, USA). This apparatus comprises a square plexiglass cage measuring 43 × 43 × 32 cm, equipped with photoresistors and sensors positioned at two heights (2.5 and 1.2 cm) along the walls. As a mouse moves and crosses the beam, the respective counter registers the movement. The disparity in counter readings reflects the spontaneous mobility of the mice.

Following the administration of the compound, each mouse was individually placed in the actimeter for 60 min. The locomotor activity of the mice was then assessed by recording the number of beam breaks at both the 30 and 60 min.

Treatment of Behavioral Research

The behavioral aspect of the study aimed to analyze the impacts of single-dose and multiple-dose administration of *S*-(+)-carvone on memory acquisition in mice via the PA test. Subsequently, an investigation was conducted to elucidate the mechanism of action of *S*-(+)-carvone by studying its influence on memory acquisition processes post-disruption induced by scopolamine.

To assess the influence of a single dose of *S*-(+)-carvone on memory processes, animals were randomly divided into four groups and received the following treatments: (1) saline solution (0.9% NaCl), (2) *S*-(+)-carvone 50 mg/kg i.p., (3) *S*-(+)-carvone 100 mg/kg i.p., and (4) *S*-(+)-carvone 150 mg/kg i.p. In order to investigate the potential mechanism of action of *S*-(+)-carvone, other animals were randomly assigned to four groups and given the following substances: (1) saline solution (0.9% NaCl) i.p., (2) scopolamine 1 mg/kg s.c., (3) *S*-(+)-carvone 50 mg/kg i.p. and scopolamine 1 mg/kg s.c., (4) *S*-(+)-carvone 100 mg/kg i.p. and scopolamine 1 mg/kg s.c. On the first day of the experiment, following the administration of the test compounds, mice underwent training in the PA test. All substances were administered 30 min prior to the pretest (assessment of memory acquisition processes). Subsequent to a 24-h interval, mice were retested in the PA test.

To evaluate the effect of multiple-dose administration of *S*-(+)-carvone on memory processes, animals were randomly divided into three groups: (1) control, (2) *S*-(+)-carvone (50 mg/kg), and (3) *S*-(+)-carvone (100 mg/kg). For six days, twice daily (at 8:30 a.m. and 6:00 p.m.), and on the seventh day in the morning, animals received the following: (1) saline solution (0.9% NaCl) i.p., (2) *S*-(+)-carvone 50 mg/kg i.p., and (3) *S*-(+)-carvone 100 mg/kg i.p. On the seventh day of the experiment, 30 min following the injection, the animals underwent training in the PA test. Subsequently, on the eighth day, the mice were subjected to a retest in the PA test.

To investigate the potential mechanism of action of *S*-(+)-carvone following multiple-dose administration, subsequent animals were randomly divided into four groups: (1) control, (2) scopolamine (1 mg/kg), (3) *S*-(+)-carvone (50 mg/kg) with scopolamine, and (4) *S*-(+)-carvone (100 mg/kg) with scopolamine. For the initial 6 days of the experiment, animals from each group received the following compounds twice daily (at 8:30 a.m. and 6:00 p.m.): (1) and (2) saline solution (0.9% NaCl) i.p., (3) *S*-(+)-carvone 50 mg/kg i.p., and (4) c *S*-(+)-carvone 100 mg/kg i.p. On the seventh day in the morning, animals received the following injections: (1) saline solution (0.9% NaCl) i.p., (2) scopolamine 1 mg/kg s.c., (3) *S-*(+)-carvone 50 mg/kg i.p. and scopolamine 1 mg/kg s.c., and (4) *S*-(+)-carvone 100 mg/kg i.p. and scopolamine 1 mg/kg s.c. On the seventh day of the experiment, 30 min after injection, animals underwent training in the PA test. On the eighth day, mice were retested in the PA test. Additionally, locomotor activity was assessed in animals assigned to groups 1, 3, and 4 following single-dose (first day) and multiple-dose (sixth day) substance administration.

Tissues Collection and Preparation

The animals were euthanized 30 min following compound administration (for single-dose treatment) or 30 min after the final dose administration (for multiple-dose treatment). Blood and brains were collected. Blood samples were centrifuged at 13,000 rpm for 10 min at +4 °C, and the resulting supernatant was collected. Brains were flushed with an ice-cold saline solution, after which the hippocampi were dissected from the brains. The collected biological samples were stored at −80 °C for subsequent analysis. Tissues were homogenized in ultrapure water (1:2 *w*/*v* for brains; 1:4 *w*/*v* for hippocampi) using a bead mill homogenizer (BeadBug 6, Benchmark Scientific, Sayreville, NJ 08872, USA) at 4000 rpm for 15 s. The homogenate was then centrifuged at 10,000 rpm for 15 min at +4 °C, and the resulting supernatant was retained for further analysis.

#### 4.2.3. Ex Vivo Assays

##### GC-MS Analysis of *S*-(+)-Carvone in Biological Material

The GC–MS system consisted of a model 7890 A series gas chromatograph coupled with Detector Agilent Technologies 5975 C inert XL MSD with Triple—Axis Detector (Agilent, IL, USA). Separation was performed on an HP–5MS column (30 m × 0.25 mm, 0.25 µm film thickness) supplied by Agilent Technologies (IL, USA). The carrier gas was 99.99% high-purity helium with a flow rate of 1.2 mL min^−^^1^. The sample volume was 1 µL. The injection port and the detector temperatures were 270 ^○^C and 280 ^○^C, respectively. The oven temperature program was initially set at 50 ^○^C for 1 min, then ramped at 3 ^○^C min^−^^1^ to 110^○^C and held for 1.0 min, then ramped at 20 ^○^C min^−^^1^ to 200 ^○^C and held for 5 min. The total run time was 31.5 min with a solvent delay of 10 min. Ionization was performed in electron impact ionization (EI) mode at 70 eV. Selective ion monitoring (SIM) was set for quantification with a dwell time of 100 ms ion^−^^1^. Data were collected using Agilent MassHunter Quantitative Analysis Software–Perlan [39]. Retention times of *S*-(+)-carvone and thymol (IS—internal standard) were 18.82 min and 21.08 min, respectively. The characteristic ions (*m*/*z*) *S*-(+)-carvone were 150, 135, 81, and for thymol (*m*/*z*) z 150, 135, 91. The final concentration of the IS was 30 µg mL^−^^1^. *S*-(+)-carvone contents were calculated from a commercial standard (Sigma-Aldrich, 124931-5ML) based on the area of the peaks obtained during chromatographic separation.

The biological material was extracted with 1 mL of saline solution (0.9% NaCl). It was then centrifuged at 12,000 rpm on a centrifuge and the supernatant was collected. To the supernatant was added 200 µL of hexane:ethyl acetate mixture (1:1) with an internal standard with a final concentration of 30 ug/mL. The sample was shaken for 10 min to extract active compounds in a vortex mixer. Then the sample was centrifuged in a high-speed cryogenic centrifuge at 12,000 rpm for 10 min and the supernatant was transferred to a 1.5 mL clean autosampler tube. One µL of the supernatant was injected into the GC–MS system for analysis.

*S*-(+)-carvone concentrations in mice were calculated using a calibration curve with the following parameters y = −0.034426x^2^ + 2.46612x+ 0.0337605.

##### Statistical Analysis

Statistical analysis of results from cell culture experiments was performed using an unpaired Student *t*-test, while statistical analysis of the data from animal studies was conducted using a one-way analysis of variance (ANOVA) followed by a post hoc Dunnett’s test, a two-way analysis of variance (ANOVA) followed by a post hoc Bonferroni’s test or a nonparametric Kruskal–Wallis ANOVA test followed by a post hoc Dunn’s test. Outliers were identified and excluded from the analysis by employing the ROUT method. The results were expressed as mean ± SD (cell culture experiments) or mean ± SEM (animal experiments). The significance level was set at *p* < 0.05.

To evaluate memory processes, a latency index (IL) was employed, calculated for each animal. IL is used to quantitatively assess PA test performance by measuring the difference between retention and training latencies, computed as follows:

IL = (TL2 − TL1)/TL1

TL1 represents the time taken to enter the dark compartment during the training phase.

TL2 denotes the duration taken to re-enter the dark compartment during retention [40,41].

## 5. Conclusions

The presented study results indicate the potential anti-neurodegenerative activity of *S*-(+)-carvone. A wide range of assays, including in vitro, in vivo, and ex vivo experiments, revealed that this monoterpene is able to inhibit BChE in vitro up to 40% and exhibits a lack of hepatotoxicity (CC_50_ > 5 mM). Important is the fact that *S*-(+)-carvone at a concentration of 0.625 mM reveals strong activity towards liver protection against the harmful effects of ROS via inhibition of oxidative stress and lipid peroxidation in in vitro conditions. Behavioral studies with mice models indicate that multiple-dose administration of the monoterpene causes improvement of memory acquisition at a dose of 100 mg/kg. Although there was a disturbance in locomotor skills, it did not affect memory processes. Significant results were obtained from GC-MS analysis of tissues (plasma, hippocampus, and remnants of the brain) collected from mice after multiple-dose administration of *S*-(+)-carvone. It has been shown that *S*-(+)-carvone is able to cross the blood–brain barrier and, importantly, it accumulates in the hippocampus (0.217 µg/mg of tissue). Considering all of the obtained results, *S*-(+)-carvone can be seen as an interesting compound characterized by various biological activities such as BChE inhibitory, hepatoprotective, non-toxic, and having an influence on memory processes. The monoterpene can be considered a promising compound in the prevention and counteraction of neurodegeneration.

## Figures and Tables

**Table 1:** BChE inhibitory activity [%] of *S-*(+)-carvone and galantamine (*n* = 3; ±SD).

Compound	Inhibitory Activity [%] for Selected Amount of Compounds ± SD			
	0.05 mg	0.025 mg	0.01 mg	0.001 mg
Galantamine *	100 ± 4.3	100 ± 3.8	100 ± 3.6	100 ± 4.1
*S*-(+)-Carvone	38 ± 1.9	40 ± 2.1	13 ± 0.62	18 ± 1.0

* galantamine activity was assumed to be 100%.

**Table 2:** The effect of *S*-(+)-carvone on locomotor activity in mice. In single-dose treatment, CV (50 or 100 mg/kg, i.p.) or saline solution (i.p.) was administered once immediately before the spontaneous locomotor activity test. In multiple-dose treatment, the same compounds were administered for 5 days, twice daily. On the sixth day, the appropriate group of mice received CV (50 or 100 mg/kg, i.p.) or saline solution (i.p.) and were then immediately placed in actimeters. Locomotor activity (number of interruptions of light beams) was recorded for 30 and 60 min. Data are presented as means ± SEM, *n* = 6–8, Dunnett’s test. * *p* < 0.05; ** *p* < 0.01; *** *p* < 0.001; **** *p* < 0.0001 vs. saline-treated control group.

	Single-Dose Treatment			Multiple-Dose Treatment		
	Control	CV 50 mg/kg	CV 100 mg/kg	Control	CV 50 mg/kg	CV 100 mg/kg
Photocell beam breaks ± SEM (30 min)	4950 ± 235.8	2606 ± 349.5 ****	1341 ± 215.2 ****	4686 ± 443.5	2704 ± 221.5 **	2803 ± 381.5 **
Photocell beam breaks ± SEM (60 min)	7938 ± 527.9	4961 ± 505.2 ***	3148 ± 408.7 ****	7721 ± 693.1	4464 ± 469.0 **	5159 ± 729.3 *

**Table 3:** Quantitative GC-MS analysis of *S*-(+)-carvone in tissues collected from mice after multiple-dose administration of the monoterpene at a dose of 100 mg/kg. Data are presented as the mean concentration ±SD [µg] in 1 mL of plasma or 1 mg of tissue from which *S*-(+)-carvone was extracted. RSD%—relative standard deviation (*n* = 3). Statistical analysis of data was performed using a *t*-test. **** *p* < 0.0001 vs. remnant of the brain.

	Tissues		
	Plasma [µg/mL]	Hippocampus [µg/mg of Tissue]	Remnant of the Brain [µg/mg of Tissue]
Average content	23.105	0.217 ****	0.045
SD	0.11	0.009	0.004
RSD%	0.51	3.96	9.46

## Data Availability

The research data are available from the authors.

## Supplementary Materials

The following supporting information can be downloaded at: https://www.mdpi.com/article/10.3390/molecules29184365/s1, Figure S1: Chromatogram of GC-MS analysis of tissues collected from mice after multiple-dose administration of *S*-(+)-carvone.
