# Supplementary material for: S-(+)-Carvone, a Monoterpene with Potential Anti-Neurodegenerative Activity—In Vitro, In Vivo and Ex Vivo Studies
Source: Molecules. 2024 Sep 13;29(18):4365. doi: 10.3390/molecules29184365 (PMC11434264; doi:10.3390/molecules29184365)
Supplement: Supplementary file 1 [file molecules-29-04365-s001.zip › molecules-3170740-supplementary.pdf]

Figure S1. Chromatogram of GC-MS analysis of tissues collected from mice after multiple-dose administration of S-(+)-carvone.

Compound Information Window

Agilent Technologies

Data Path D:\Masshunter\GCMS\1\data\180424_carvone_myszy

Data File carvon_osocze_2_180424.D

Acq Operator

Acq. Date-Time 4/18/2024 5:51 PM

Instrument Name GCMS

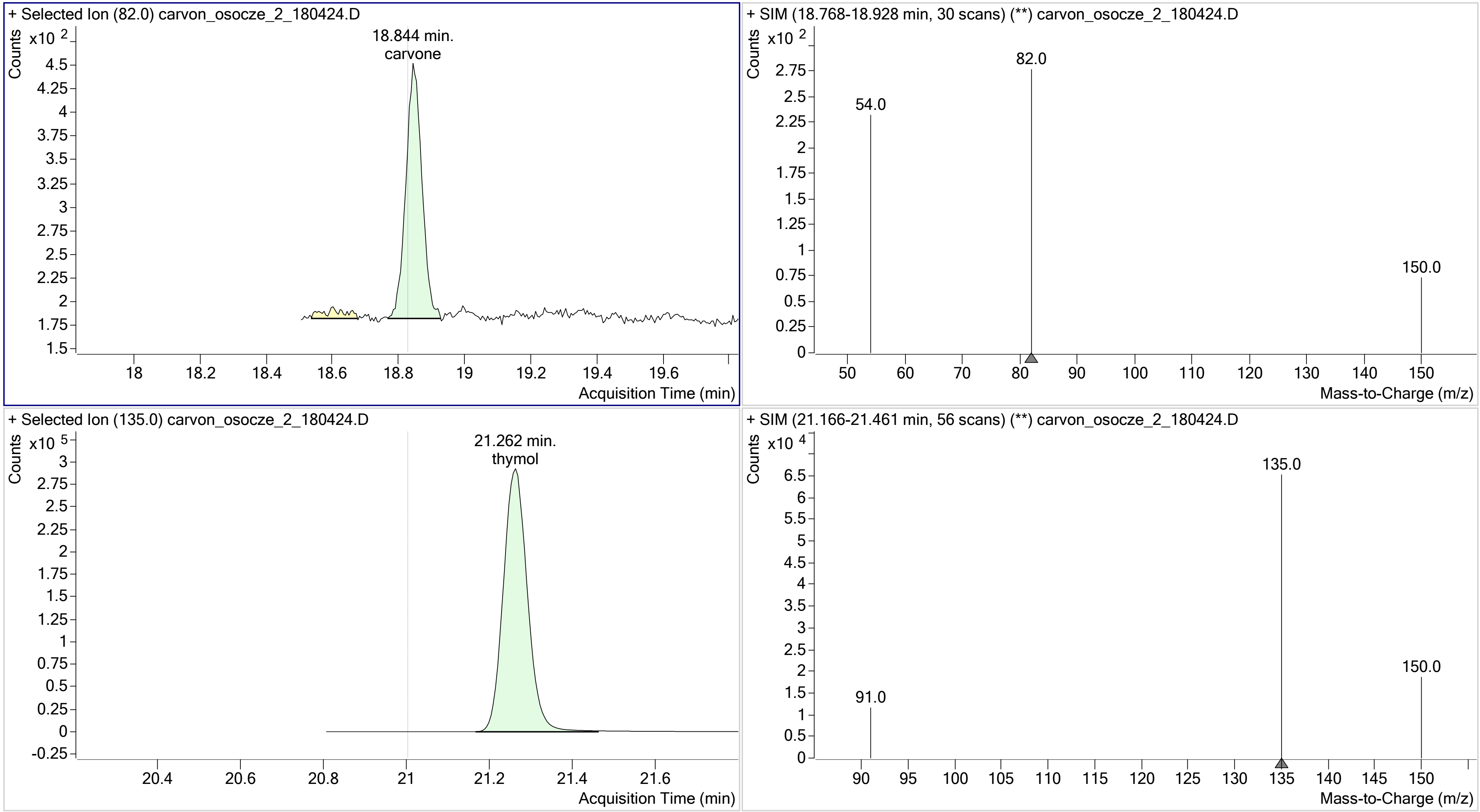

Generated at 9:14 AM on 8/19/2024
